# Unresolved trauma in mothers: intergenerational effects and the role of reorganization

**DOI:** 10.3389/fpsyg.2014.00966

**Published:** 2014-09-01

**Authors:** Udita Iyengar, Sohye Kim, Sheila Martinez, Peter Fonagy, Lane Strathearn

**Affiliations:** ^1^Department of Pediatrics, Children's Nutrition Research Center, Baylor College of MedicineHouston, TX, USA; ^2^Attachment and Neurodevelopment Laboratory, Children's Nutrition Research Center, Baylor College of MedicineHouston, TX, USA; ^3^Research Department of Clinical, Educational, and Health Psychology, University College LondonLondon, UK; ^4^Menninger Department of Psychiatry and Behavioral Sciences, Baylor College of MedicineHouston, TX, USA; ^5^The Meyer Center for Developmental Pediatrics, Texas Children's HospitalHouston, TX, USA

**Keywords:** unresolved trauma, intergenerational transmission, attachment, mother-infant, reorganization

## Abstract

A mother's unresolved trauma may interfere with her ability to sensitively respond to her infant, thus affecting the development of attachment in her own child, and potentially contributing to the intergenerational transmission of trauma. One novel construct within the Dynamic Maturational Model of Attachment and Adaptation (DMM) coding of the Adult Attachment Interview (AAI) is “reorganization,” a process whereby speakers are actively changing their understanding of past and present experiences and moving toward attachment security. We conducted a study of mothers with unresolved trauma, exploring their own attachment classification, attachment outcomes of their children, and the potential effects of reorganization on child attachment. Forty-seven first-time mothers participated in the AAI during pregnancy, and returned with their child at 11 months to assess child attachment using the Strange Situation Procedure. Mothers with and without unresolved trauma were compared. We found that mothers with unresolved trauma had insecure attachment themselves and were more likely to have infants with insecure attachment. However, the one exception was that all of the mothers with unresolved trauma who were reorganizing toward secure attachment had infants with secure attachment. These preliminary findings suggest that mothers who are reorganizing may be able to more sensitively respond to their child's cues, contributing to the development of secure attachment. While our results need to be replicated in a larger cohort, this study is the first to explore the construct of reorganization and its potential relationship with child attachment. If confirmed in future studies, it may provide clinical insight into the intergenerational transmission of insecure attachment within the context of unresolved trauma.

## Introduction

Attachment theory emphasizes the quality of one's early relationships and how this affects aspects of later functioning, wherein the attachment relationship between mother and infant ensures infant survival, as well as optimal social, emotional, and cognitive development (Insel and Young, 2001; Sroufe, 2005). Mothers who are sensitive to their children's signals, who are emotionally available, perceptive, and responsive to their infants' needs and mental states, have infants who are more likely to be securely attached (Siegel, 1999). Empirical research has documented patterns of sensitive responsiveness leading to secure attachment across generations, as observed in numerous studies utilizing the Adult Attachment Interview (AAI; George et al., unpublished manuscript), and measures of infant attachment, such as the Strange Situation Procedure (Ainsworth et al., 1978). From both cross-sectional and prospective longitudinal studies, the AAI has been widely used and shown to reliably predict maternal behavior patterns, development of infant attachment (van IJzendoorn, 1995; van IJzendoorn and Bakermans-Kranenburg, 2009), and infant social and emotional development (Sroufe, 2005). For instance, a meta-analysis of 13 studies examined the correspondence between mother and infant attachment, indicating a high concordance rate (75%) between secure attachment in mothers and infants (van IJzendoorn, 1995). Our previous study suggested a similar pattern of transmission between mothers and infants, with secure attachment classifications between mother and child matching 73.4% of the time (Fonagy et al., 1991; Shah et al., 2010). Whether the data are examined prospectively, retrospectively, or concurrently, these studies have provided evidence for the validity and cross-generational stability of secure attachment.

Patterns of insecure attachment across generations have also been widely substantiated, particularly when exploring the impact of unresolved trauma of parents in relation to their children. Main and Hesse (1990) first found an association between the adult attachment pattern of an adult who lost her parent in childhood, and the subsequent transmission of insecure (specifically, disorganized) attachment to the infant, based on AAI and SSP. A follow-up study by Ainsworth and Eichberg (1991) found that in the AAI, a mother's discourse regarding a loss that she faced in her childhood was also related to infant disorganization. Schuengel et al. (1999) similarly found that mothers with insecure attachment who had an unresolved trauma or loss of an attachment figure tended to have children with insecure attachment themselves, more so than if the mother had an unresolved trauma or loss with *secure* attachment. Infants of traumatized mothers also appear to find it difficult to seek comfort when distressed, and demonstrate frightened and alarmed behavior when in the presence of their traumatized mothers (Hesse and Main, 1999; Lyons-Ruth et al., 1999; Madigan et al., 2006). The occurrence of an unresolved trauma or loss may therefore impair a mother's ability to respond sensitively and effectively to her infant's needs, possibly intensifying the behavior of her child when frightened or alarmed, and increasing the risk of insecure attachment.

Schwerdtfeger and Goff (2007) explored the relationship between trauma history and symptomatology in expectant mothers, and subsequent development of attachment and bonding to their unborn children, in an effort to understand the possible intergenerational transmission of trauma. They found that interpersonal trauma had negative effects on prenatal attachment, such that disturbances in the caregiver-child relationship were associated with the maternal perception of the “child as threat” (Schechter et al., 2004, p. 321). Unresolved trauma or loss may interfere with a mother's expectations and perceptions of her child, as well as her ability to sensitively respond, thus compromising the development of secure attachment in her infant.

On a neurobiological level, our other recent study utilizing functional magnetic resonance imaging (fMRI) demonstrated the impact of unresolved trauma on brain response when mothers viewed sad face images of their infants (Kim et al., 2014). In this sample, mothers who were classified as having unresolved trauma or loss displayed reduced activation in the amygdala, a key neural structure involved in emotion processing (Sander et al., 2003; Ewbank et al., 2009; Adolphs, 2010) which is susceptible to both structural (Rauch et al., 2000) and functional change (Rauch et al., 2000; Protopopescu et al., 2005; Williams et al., 2006a,b) in response to trauma. This blunted amygdala response was seen only when these mothers viewed their own infant's distressed face, and not that of unknown infants. This may reflect traumatized mothers' disengagement from their infants' distress, and could contribute to the transgenerational transmission of trauma. Unresolved trauma may therefore interfere with a mother's expectations and perceptions of her child, her ability to sensitively respond to her infant, and associated neural responses to infant emotional cues, thus compromising the development of secure attachment in her infant.

While the intergenerational transmission of secure and insecure attachment has been well established, our previous study (Shah et al., 2010) also found an interesting reversal of attachment strategies within *insecure* attachment across generations, whereby mothers with preoccupied attachment tended to have infants who used an avoidant strategy, and vice-versa. This pattern of attachment strategy reversal between parent and child has also been observed in other normative samples (Hautamaki et al., 2010), as well as in families with a history of maltreatment who are at high risk for insecure attachment (Crittenden et al., 1991; Farnfield et al., 2010). To further understand the transmission of insecure attachment being transmitted across generations, with an emphasis on unresolved trauma, we chose to utilize the Dynamic Maturational Model of Attachment and Adaptation (DMM) (see Farnfield et al. (2010) for a detailed description of the various DMM measures). The DMM views attachment as an array of self-protective strategies organized around danger (Crittenden, 1992, 1995, 1997, 2000). In the DMM, patterns of attachment vary dimensionally (rather than categorically) to encompass a broader scope of attachment-related characteristics, or strategies. According to the DMM, an individual with balanced or secure attachment uses cause and effect contingencies learned from previous experiences (defined here as “cognition”) and a range of positive and negative emotions (defined here as “affect”), while taking a variety of perspectives when drawing conclusions (one's own and others'), and demonstrating thoughtful and reflective integration when recalling childhood experiences and relationships. When faced with adversity or situations in which attachment figures fail to protect or comfort, an individual may develop self-protective strategies leading to non-balanced, or insecure attachment.

At times, a life event or circumstance can be psychologically traumatic for an individual, such as instances of abuse or loss of an attachment figure. Exposure to danger or loss may lead to several possible outcomes. One is that an individual may garner new information from the situation and context to integrate the experience, resulting in adaptation and a newfound understanding that helps the individual to avoid future danger. These individuals would be considered “resolved” with regard to the trauma or loss. In another scenario, individuals experiencing trauma or loss may either retain too much information about the traumatic event or dismiss the importance of it, in ways that are maladaptive to future processing of information (Crittenden and Landini, 2011). For example, an adolescent girl may experience a hypervigilant fearful response when in close contact with a male schoolteacher who reminds her of a childhood sexual predator. Alternately, this same adolescent may ignore relevant information and memories to place herself at increased risk of further sexual abuse. A lack of resolution of trauma suggests that the event has not been adequately integrated or reflected upon, leaving potential for future risk. These individuals will be classified, on the basis of their AAI, as having unresolved trauma or loss, may be at risk for developing later psychosocial problems (Cicchetti and Toth, 1999; Easterbrooks et al., 2000), and most relevant to our study, may be more likely to propagate the cycle of insecure attachment to include their own offspring.

Despite evidence of the continuity of both secure and insecure attachment across generations, this is not always the case. Bowlby (1973) himself understood that change in attachment strategy is possible, and can be associated with major life changes or a series of events across the lifespan. Some individuals with patterns of insecure attachment (transmitted across generations), who have experienced harsh parenting or adverse life events, are able to overcome the effects of these experiences and demonstrate balanced integration. These individuals are labeled as “earned secure” by some theorists (Pearson et al., 1994; Roisman et al., 2002). Earned secure individuals are thought to have interrupted the intergenerational cycle by demonstrating emotional resilience (Fearon et al., 2010).

Less explored is the process by which those with insecure attachment actively change their understanding of past and present experiences, often in the direction of greater balance and resolution (Crittenden and Landini, 2011). This construct is referred to as “reorganization” in the DMM, and is based on clinical observations from within AAIs (Crittenden, 1990, 1995). Whilst it is not necessary for an individual to experience danger or trauma before undergoing reorganization, the experience and resolution of danger can at times in an alteration in mental processing and behavior, leading to a change in attachment strategy (Crittenden, 2008). The modified AAI coding scheme aims, through the process of discourse analysis, to identify individuals who are undergoing reorganization. Although not fully secure or balanced in attachment terms, reorganizing individuals may exhibit mental resilience to buffer the effects of adversity or unresolved trauma as a result of maturation or changing circumstances (Landa and Duschinsky, 2013), which may have positive effects on the security of their progeny. Little is known about those individuals who have unresolved trauma or loss but who are in the dynamic process of altering their attachment strategy toward security. The process of reorganizing may alter the transmission of insecure attachment from one generation to the next, an idea that has important clinical implications but has received little attention in the literature.

The transmission of secure and insecure attachment across generations, as well as the contribution of earned security in this process, has been previously explored and established (Fonagy et al., 1991; van IJzendoorn, 1995; Hesse and Main, 1999; Schuengel et al., 1999; Lyons-Ruth et al., 2005; Shah et al., 2010). In the present study, we sought to understand how the process of reorganization might positively impact the transmission of attachment across generations. This is the first paper to explore the concept of reorganization within the context of the intergenerational transmission of attachment, thereby adding to the validity of a novel and clinically relevant attachment-related construct. The goal of this study was to examine associations between unresolved trauma and mother and child attachment, while testing whether reorganization is associated with more secure child attachment. Consistent with previous studies (van IJzendoorn, 1995; Hesse and Main, 1999; Schuengel et al., 1999; Lyons-Ruth et al., 2005), we hypothesized that mothers with unresolved trauma would be more likely to have insecure attachment (hypothesis 1), as well as children with insecure attachment (hypothesis 2). In addition, we tested two hypotheses relating to change in attachment style. We predicted that mothers with unresolved trauma (Utr) who were reorganizing toward security would be more likely to have securely attached children (hypothesis 3). We also predicted that maternal reorganization would explain incremental variance in child attachment security above and beyond that accounted for by maternal Utr (hypothesis 4). While predicting that unresolved trauma would be associated with insecure child attachment, we further hypothesized that maternal reorganization would add to this model to better predict the outcome.

## Materials and methods

### Participants

Sixty-seven first time mothers were recruited during their third trimester of pregnancy and followed-up 11 months postnatally, with 47 mothers completing both the AAI and the SSP. Mothers were recruited through advertisements posted in prenatal clinics, magazines, on billboards and via the Internet. Potential subjects were excluded if they were on psychotropic medications, were using cigarettes during pregnancy, or were left-handed (due to the neuroimaging component of the study). The Institutional Review Board at Baylor College of Medicine approved the research protocol, and all subjects provided written informed consent.

### The adult attachment interview

The Adult Attachment Interview (AAI; Crittenden and Landini, 2011; George et al., unpublished manuscript) is a semi-structured 1½–2 h-long interview comprised of questions which highlight childhood relationships with attachment figures, usually parents. A modified version of the AAI was chosen because of its expanded questions that contain more direct probes into potential life traumas and losses (Shah and Strathearn, 2014), and validation from previously published data (Strathearn et al., 2009). Through analysis of the discourse, an overall attachment strategy is determined [labeled in the Crittenden model as A, B, or C, paralleling the infancy model of attachment from the Strange Situation Procedure (SSP)], along with discourse modifiers, including “unresolved trauma or loss,” and those meeting the classification of “reorganization.”

We chose to measure attachment during pregnancy using a longitudinal design to exclude the possibility that the infant's temperament or mother–infant interaction patterns might influence the way the mother describes her own attachment experiences. Each interview was digitally recorded and transcribed. In order to maintain confidentiality, all identifying information was changed. To categorize the mothers' adult attachment strategies, the transcripts were coded blindly by reliable raters according to the Dynamic Maturational Model (DMM) of Attachment and Adaptation. Adult classifications were not revealed until the completion of the study, and 50% of the transcripts were double coded to ensure reliability. Discrepancies were resolved through conferencing between coders, and the intraclass correlation for the unresolved trauma classification was 0.86.

#### Coding unresolved trauma

As conceptualized by Bowlby (1982) and Crittenden and Landini (2011), the notion of unresolved loss was considered to be a subcategory of trauma, and is hereafter referred to under the term “unresolved trauma.” Utr was coded when examining especially frightening or dangerous experiences that continued to affect the speaker's thoughts, feelings, or behavior. The coding of Utr may include how the incident was mentioned (for example, if omitted when relevant, or introduced more often than necessary in a preoccupying way, etc.), changes in discourse during discussion of the trauma or loss (such as errors in tense or lapses in grammar), as well as the overall consideration of relationships and effects of the traumatic incident.

#### Coding reorganization

In the coding process, a reorganizing speaker is identified as actively changing their perception and understanding of their experiences, both past and present. Reorganizing speakers make reflective and evaluative statements indicative of mental balance, incorporate new information to achieve a new understanding of situations, consider alternate perspectives, and achieve a cooperative relationship with the interviewer to find meaning in their history (Crittenden and Landini, 2011). There are slips into the dominant pattern of insecure attachment, and some level of unresolved incoherence noted in the discourse, but the reorganizing speaker attempts to reach an integrative conclusion about their situation. The coding of reorganization includes a list of hallmark features, with a specific number of criteria needed before reaching this classification. For a complete list of reorganizing criteria, see Supplementary material.

### Strange situation procedure

The SSP (Ainsworth et al., 1978) is a standard procedure used to classify infant attachment between 11 and 15 months of age (Farnfield et al., 2010). Three reliable coders blindly coded the SSP videos, using DMM coding criteria. The kappa on a 4-way classification of SSPs ranged from 0.47 to 0.59 (Shah et al., 2010) and where discrepancies existed, the final classification was determined through conferencing.

### Self-report questionnaires

Mothers completed a battery of questionnaires, including the Adult Temperament Questionnaire (ATQ; Rothbart et al., 2000), Personality Development Questionnaire (PDQ; Hyler et al., 1992), Beck Depression Inventory (BDI; Beck et al., 1996), Parenting Stress Index (PSI; Abidin, 1995), and the Infant Behavior Questionnaire-Revised (IBQ; Gartstein and Rothbart, 2003). The ATQ, IBQ, and PSI were completed during a separate visit (approximately 7 months postpartum). The Bayley Scales of Infant and Toddler Development, Screener, Third Edition (Bayley III; Bayley, 2006) was completed when infants were 11 months of age. For the details of all the instruments, please refer to Shah et al. (2010).

### Procedures

In the last trimester of pregnancy, mothers participated in the modified version of the AAI, at which time socio-demographic information and behavioral measures were also collected. The mothers returned with their child at 11 months postpartum, at which time the SSP was conducted, as well as an assessment of infant development using the Bayley-III.

### Analysis plan

The two groups of mothers (Utr vs. No Utr) were compared in terms of demographic and questionnaire measures using Chi-square and *t*-tests for categorical and continuous data, respectively. Hypotheses 1–3 were tested using Chi square and Fisher's exact tests, examining associations between maternal attachment security, maternal reorganization, and infant attachment security for mothers with and without Utr. Hypothesis 4 was tested using a likelihood-ratio chi-square test, examining a change in model fit when maternal reorganization was added to the model (which contained maternal Utr as the sole predictor of infant attachment security). All analyses were conducted using SPSS version 21 and STATA/SE version 12.1.

## Results

### Study participants

Socio-demographic information and self-report measures are presented in Table 1. In addition to the overall AAI classification (secure vs. insecure), the mothers were dichotomized into having unresolved trauma or loss (Utr, *n* = 18), vs. no unresolved trauma or loss (No Utr, *n* = 29). Mothers with Utr were significantly older than mothers with no Utr, and 87% of mothers with Utr identified themselves as white compared to 52% of mothers with no Utr. The groups did not differ significantly in any other socio-demographic variable, including income level, marital status or IQ. In behavioral measures, mothers with Utr scored their infants lower on the “approach” subscale of the IBQ, but there were no other differences between groups in any measures of adult temperament, personality disorder, parenting stress, depression, or infant development.

**Table 1:** **Demographic characteristics of mothers with or without unresolved trauma (Utr) (*N* = 47)**.

**Variable**	**No Utr (***N*** = **29**)**	**Utr (***N*** = **18**)**
Age of mother (in years) ± SD	27.98 ± 4.2	30.22 ± 4.8^*^
**Marital status, N (%)**		
Married	20 (69)	16 (89)
Not married	9 (31)	2 (11)
**Maternal race, N (%)**		
White	15 (52)	15 (83)^*^
Non-white	14 (48)	3 (17)
**Income, N (%)**		
<$30,000/year	9 (31)	4 (22)
$30,001–70,000	9 (31)	5 (28)
>$70,000	11 (38)	7 (39)
Not reported	0	2 (11)
**Mother-infant separation, N (%)**	**(*N* = 26)**	**(*N* = 18)**
≤20 h/week	13 (50)	8 (44.4)
≥20 h/week	13 (50)	10 (55.6)
Maternal IQ (predicted from WTAR), M ± SD^a^	109.56 ± 9.9	109.72 ± 7.3
**Maternal depression (BDI), N (%)^b^**	**(*N* = 29)**	**(*N* = 17)**
Minimal	24 (83)	15 (88)
Mild to moderate	5 (17)	2 (11)
**Personality Disorder Screen (PDQ)^c^**	**(*N* = 29)**	**(*N* = 17)**
Total score, mean ± SD	18.9 ± 10.3	18.5 ± 10.1
**Parenting Stress Index (PSI), M ± SD^d^**	**(*N* = 25)**	**(*N* = 14)**
Child domain total score	91.7 ± 13.1	90.4 ± 14.7
Parent domain total score	111.0 ± 23.7	112.7 ± 19.6
Total stress score	202.8 ± 28.7	195.3 ± 38.7
**Temperament of Mothers (ATQ), M ± SD**	**(*N* = 28)**	**(*N* = 17)**
Effortful control	4.6 ± 0.6	4.5 ± 0.8
Negative affect	3.9 ± 0.7	3.9 ± 0.7
Extraversion/Surgency	4.9 ± 0.6	4.8 ± 0.6
Orienting sensitivity	5.1 ± 0.7	5.1 ± 0.6
**Infant development (Bayley), mean raw score ± SD**	**(*N* = 29)**	**(*N* = 17)**
Cognitive	17.6 ± 1.5	17.4 ± 2.0
Receptive communication	14.7 ± 2.2	14.0 ± 2.1
Expressive communication	13.9 ± 1.9	13.5 ± 1.9
Fine motor	15.7 ± 1.8	15.9 ± 2.0
Gross motor	18.2 ± 1.3	17.3 ± 1.9
**Infant temperament (IBQ), M ± SD**	**(*N* = 27)**	**(*N* = 17)**
Approach	5.8 ± 0.8	4.9 ± 1.5^*^
Vocal reactivity	5.2 ± 0.8	5.0 ± 0.9
High intensity pleasure	6.0 ± 0.5	6.1 ± 0.7
Activity level	4.9 ± 0.8	4.7 ± 0.9
Perceptual sensitivity	4.8 ± 1.2	4.3 ± 1.0
Distress to limitations	3.9 ± 0.6	3.8 ± 0.8
Fear	2.5 ± 0.8	2.8 ± 1.5
Low intensity pleasure	5.0 ± 1.3	5.5 ± 0.9
Cuddliness	5.5 ± 0.6	5.8 ± 0.7
Duration of orienting	4.4 ± 1.0	3.9 ± 1.1

* p < 0.05.

BDI, Beck Depression Inventory-II; PDQ, Personality Disorder Questionnaire-4+; PSI, Parenting Stress Index; ATQ, Adult Temperament Questionnaire—Short Form; IBQ, Infant Behavior Questionnaire.

a Maternal Full Scale IQ was estimated from the Wechsler Test of Adult Reading (WTAR).

b BDI-II score of 0–13 indicates minimal depression, 14–19 indicates mild depression, and 20–28 indicates moderate depression.

c PDQ-4+ total score of ≥50 is highly suggestive of DSM-IV personality disorder.

d I Total Stress Score of <260 is considered normal range.

#### Hypothesis 1. mothers with Utr are more likely to have insecure attachment

Consistent with our prediction, 100% of the mothers with Utr had insecure attachment themselves, compared with only 25% of mothers without Utr (Fisher's exact test: *p* < 0.001; Table 2).

**Table 2:** **Adult and infant attachment classifications in mothers with and without unresolved trauma (Utr)**.

**Mothers with Utr**	**Mother attachment^a^**		**Infant attachment^b^**	
	**Secure**	**Insecure**	**Secure**	**Insecure**
Yes, N (%)	0 (0)	18 (100)	4 (22)	14 (78)
Adjusted residual	−5.1	−5.1	−2.2	2.2
No, N (%)	22 (76)	7 (24)	16 (55)	13 (45)
Adjusted residual	5.1	−5.1	2.2	−2.2

a χ^2^ = *25.672*, df = 1, p < 0.001, K = 0.707;

b χ^2^ = *4.933*, df = 1, p = 0.026, K = 0.301.

#### Hypothesis 2. mothers with Utr are more likely to have infants with insecure attachment

We found that over 75% of mothers with Utr had insecurely attached infants, compared with only 45% of mothers without Utr (Fisher's exact test: *p* = 0.036; Table 2).

#### Hypothesis 3. mothers with Utr who are reorganizing toward security are more likely to have a securely attached infant

Of the mothers who had unresolved trauma (*n* = 18), four (22%) were reorganizing toward secure attachment (Table 3A). On examining the possible intergenerational effect of reorganizing attachment, we found that all four of these reorganizing mothers had children with secure attachment, while none of the non-reorganizing mothers had secure children (Fisher's exact test: *p* < 0.001; Table 3B).

Table 3**Infant attachment security predicted by reorganization in mothers with unresolved trauma (Utr)**.

**Table:** 

**(A)**			
**Utr status**	**Reorganization status**		**Total**
	**Reorganizing**	**Not reorganizing**	
Utr, N (%)	4 (22)	14 (78)	18 (100)
No Utr, N (%)	0 (0)	29 (100)	29 (100)

χ^2^ = *7.044*, df = 1, p = 0.008, K = 0.261.

**Table:** 

**(B)**		
**Reorganizing status of Utr mothers**	**Infant attachment**	
	**Secure**	**Insecure**
Reorganizing, N (%)	4 (100)	0 (0)
Adjusted residual	4.2	−4.2
Nor reorganizing, N (%)	0 (0)	14 (100)
Adjusted residual	−4.2	4.2

χ^2^ = 18.000, df = 1, p < 0.001, K = −0.528.

#### Hypothesis 4. maternal reorganization will explain incremental variance in infant attachment security above and beyond that accounted for by maternal Utr

Compared to the model containing maternal Utr as the sole predictor of infant attachment security [β_Utr_ = 0.330, Model *R*^2^ = 0.105, *F*_(1, 45)_ = 5.28, *p* = 0.026], adding maternal reorganization led to a significant increase [LR χ^2^(1) = 16.93, *p* < 0.0001] in model fit [β_Reorganization_ = −1, Model *R*^2^ = 0.376, *F*_(2, 44)_ = 13.24, *p* < 0.001].

## Discussion

This is the first study to examine the construct of “attachment reorganization” as measured by the DMM and its effect on children's attachment security within the context of intergenerational transmission of trauma and attachment. As expected, mothers with unresolved trauma were universally classified as having insecure attachment themselves, and were less likely to have children who were securely attached when compared to mothers with no unresolved trauma. Using the Crittenden's Dynamic Maturational Model of Attachment and Adaptation, these results reaffirm the role of unresolved trauma in the intergenerational transmission of insecure attachment (van IJzendoorn, 1995; Hesse and Main, 1999; Schuengel et al., 1999; Lyons-Ruth et al., 2005). Most importantly, individuals who were identified as reorganizing toward secure attachment before the birth of their child were more likely to have securely attached children at 11 months postpartum. Although preliminary, this study suggests that the construct of “reorganization” may help us better predict attachment outcomes in the children of mothers with unresolved trauma.

Except for maternal age and race variables, mothers with and without Utr could not be readily distinguished by other socio-demographic factors, self-report measures of temperament, personality traits, psychopathology risk, or infant development. It was interesting to note that, in the Infant Behavior Questionnaire, mothers with Utr rated their infants as having less “approach” behavior, which subscale included infant “excitement” and “positive anticipation of pleasurable activities” (Gartstein and Rothbart, 2003). The diminished approach behavior in infants of mothers with Utr is consistent with literature showing that infants of traumatized mothers tend to be more inhibited or even fearful in their mother's presence (Hesse and Main, 1999; Lyons-Ruth et al., 1999).

### Unresolved trauma and reorganization

In considering the implications of our findings, it is important to note that coding unresolved trauma from the AAI transcript is based less on the description of the traumatic event and more on how the experience impacts the individual's current functioning and evaluation of the event (Crittenden and Landini, 2011). In the DMM coding of the AAI, the presence of Utr in an individual suggests maladaptive information processing. Yet, what is it about *reorganizing* mothers in particular that could positively impact their children's attachment status when mothers themselves have unresolved trauma? One way to differentiate a reorganizing and a non-reorganizing speaker, both of whom have a classification of unresolved trauma, is to explore the discourse surrounding the event, as well as their integration of the event with respect to current functioning, based on their AAIs. To illustrate the difference, Nina and Marlene (names have been changed) are examples of speakers in our sample with similar cognitive-driven attachment strategies as well as unresolved trauma. Both also presented with unresolved traumas regarding frightening incidents with their parents, specifically their fathers. Marlene was classified as having insecure attachment but was in the process of reorganizing toward security, and had a child with secure or balanced attachment, while Nina and her child both had insecure attachment.

#### Example 1. marlene describes her father as “frightening,” and relates an experience in which her father caused a second-degree burn in teaching her a lesson about overcoming her fears

… the water started to boil and I, the steam was coming out from the sides of the, top of the, the pot, and I was scared of the steam. I was scared to take the top off… My dad said in his very slow, methodical way “just take the top off.” “No, but there's steam ah ah.” … and very slowly walking over to me and taking his hand over my hand and, you know, … slowly, deliberately, on purpose, and unknowingly, he pressed the tips of my fingertips down on the top of, of the pot top (nervous laugh), which was metal um, and I started screaming as my finger tips are being burned off …. … Not that he would ever intentionally hurt me…

#### Example 2. Nina describing her father as being “volatile,” and provided two separate examples of her frightening experiences

Nina: … I'd run as far as I could go thinking I was going to get away with, get away from them, which probably wasn't very far, and then as far as, and then I could get as far as I could go, I would drop to the ground so the butt was close to the ground, but you know like they do in a tornado drill for a little kid? Run as far as away, realize they were catching me, drop to the ground so that the butt's covered, cover the head up, so the only thing out is the back. So if they're going to spank me, they have to spank my back (chuckle). That's… so.. and then uhhhh… you know, I don't really remember ever being really… hurt. I never really got.. seriously hurt.”

Interviewer: Well, did you ever feel very frightened or unsure that you were safe?

Nina: The teeth pulling thing for sure. That terrified me for some reason. So… He, he… you know I still think the dentist must have told him to do it for some reason. I don't really know why he did it. Two people had to hold me to make it possible. So, uh, like my mom and my dad and, you know, he's got these pliers (chuckle). So I remember… being… uh… you know, they, they came in and woke me up… to. … so… I mean ultimately, all that happened was a loose tooth got pulled. You know, not a big deal.

When describing the traumas, there are similar features of Marlene and Nina's discourse that rendered them as unresolved, particularly the slips into present tense (e.g., “and very slowly *walking* over to me and *taking* his hand over my hand”; “I started screaming as my finger tips *are* being burned off”; and “*run* as far away realize they were catching me, *drop* to the ground… *cover* the head up), and false positive laughter intended to minimize the severity of the trauma (“he pressed the tips of my fingertips down on the top of, of the pot top [*nervous laughter*],” “so if they are going to spank me, they have to spank my back [*chuckle*]”). These discourse markers indicate a preoccupying unresolved trauma, whereby the present tense brings the threatening event closer in time and space, possibly distorting when and where they may be in danger (Crittenden and Landini, 2011), and allowing the speaker to maintain a vigilant awareness of all the details. The false positive laughter after the dangerous event serves to minimize discomfort. At the same time, there is a dismissing component to the traumas, with both speakers suggesting that the events were unimportant, and that the parents were not responsible for any harm (e.g., “not that he would ever intentionally hurt me”; “you know I don't really remember ever being really… hurt. I never really got… seriously hurt”; and “so… I mean ultimately, all that happened was a loose tooth got pulled. You know, not a big deal”). This discourse may result in decreased accuracy in predicting future danger, thus placing speakers at greater risk of misinterpreting or omitting future danger cues (Crittenden, 2008). This is relevant when considering decreased maternal attentiveness and sensitivity to infant cues, both at times of safety and danger, which may compromise the quality of parent-child interaction.

#### Examples of reorganization and non-reorganization in discourse

When comparing their overall discourse in the interview, we note how Marlene is reorganizing toward secure attachment, while Nina is still maintaining a compulsive cognition-driven insecure attachment strategy that revolves around inhibiting her own feelings and seeking to please others. In the final integrative section of the interview, we note Nina's difficulty in reflecting on how her childhood experiences may have hindered her development:

Nina (non-reorganizing): “I think that my, you know… I, I, I don't know. There's a balance… I mean they kind of just let us, let me particularly I guess, it seems more than even my brother, I'm… make a lot of the decisions for ourselves probably because I was so volatile?”

When asked about what she wished to do differently with her own children:

“Definitely no physical punishment. Like, uh.. I, I'm not saying that a kid doesn't deserve a spanking. I actually still believe in punishing, but you don't ever do it out of anger…. I mean, we've actually talked about that, my husband and I because he knows, you know, he knows kind of how things were done at my house and I think… he probably got slapped once by his mom and deserved it (chuckle).”

Nina is unable to articulate the effects that the parental punishment had on her, and instead describes herself as “volatile,” the same word she had used to describe her father. She is aware of the impact of her punishment at the hands of her parents, yet she is distancing in the way that she describes it, saying that “you” don't ever do it out of anger, and that her husband knows “kind of how things were done at my house,” and then enacts her pattern of chuckling after abuse is mentioned, much like when she described her own circumstances as a child. The final section of the interview is intended to illicit reflective thought and perspective about past experiences, yet Nina is still unable to accurately reflect on how her parent's abuse impacted her.

Marlene, who was reorganizing toward security and whose child had secure attachment, also experienced a very frightening event with her father as a young child. Yet she is able to reflect on the good and the bad, without distancing herself or blaming her parents excessively.

When asked about her experience as an adolescent:

Marlene: “I didn't like how strict (my parents) were and, you know… they wanted to teach us these values and… I didn't see how that was benefitting me at all and, and now I do see how it has benefited me and I, I'm glad for it but, man there were times when I hated them for it.”

When asked about what she would do differently from what her parents did:

Marlene: “I probably won't be quite as strict as my mom was, as my parents were. I'm, I hope not but (laugh), I slip into it. I don't know.”

Marlene is able to comment openly about an aspect of her parents' behavior that she wishes to change, but with open and fluent discourse, while displaying her ability to self-monitor, noting that she may sometimes slip into her old patterns of behavior. Marlene is reorganizing toward secure attachment based on her discourse, but still retains some aspects of insecure attachment. However, a reorganizing speaker such as Marlene may be more able to integrate the signals given by her infant without distortion, particularly in times of danger, with a balanced overall understanding of the experience. A non-reorganizing speaker with unresolved trauma, such as Nina, is still at risk of falsifying, distorting, or changing the meaning of her infant's signal in times of danger, which may compromise the dyadic interchange between herself and her child. As demonstrated in our recent neuroimaging paper (Kim et al., 2014), mothers with unresolved trauma do show an atypical brain amygdala response when processing their own infant's distress signals, which may result in misattunement during moments of infant distress.

### Reorganization and clinical implications

Our preliminary results suggest that reorganization may be associated with child attachment security, despite mothers themselves being insecurely attached. Bowlby himself (1984) believed that current attachment representation is formed on the basis of early attachment experiences, but is also influenced by later relationships or changing circumstances. This concept is particularly important in a therapeutic setting, where therapists are facilitating change in their patients' intrapsychic and interpersonal relationships. While we were not able to determine the precipitating events/people (such as spouse, therapist, birth of a child, etc.) which led to reorganization in our sample, our data suggest that this construct should be further evaluated for its potential role in modifying the cycle of intergenerational transmission of attachment.

### Limitations and future directions

A major limitation of the study the small number of mothers who were identified as having reorganizing attachment: out of the 47 mothers who completed both the AAI and SSP, only 4 mothers had both unresolved trauma and reorganizing attachment. Our findings regarding reorganization reported here are therefore preliminary and need replication in a larger study. Future studies will explore the concepts of unresolved trauma and reorganization within a larger population sample. Furthermore, additional factors such as parental sensitivity and interactive behavior, and infant temperament, may contribute to the development of secure child attachment (van IJzendoorn and Bakermans-Kranenburg, 2004). While our results provide some insight into how a mother with reorganizing attachment may promote the development of secure attachment, the ways in which maternal reorganizing attachment interacts with other factors also remains to be explored. We are currently exploring reorganization in a larger population of women with substance abuse disorders, where unresolved trauma is more prevalent.

## Conclusions

A significant proportion of clinical work revolves around helping patients in the resolution of childhood trauma. Our study reaffirms that unresolved trauma is associated with insecure attachment in both mothers and their offspring, while providing the first preliminary evidence that attachment reorganization may lessen the risk of insecure attachment in the offspring.

While preliminary, this is the first empirical examination of the construct of attachment reorganization, which may ultimately be relevant in clinical settings in which attachment trauma is often a prominent focus of treatment. This study adds to the current literature by providing initial empirical support for the construct of reorganization. With further examination and validation, this construct has the potential to significantly enrich the field's understanding of the intergenerational transmission of attachment.

### Conflict of interest statement

The authors declare that the research was conducted in the absence of any commercial or financial relationships that could be construed as a potential conflict of interest.
