# Supplementary material for: Unresolved trauma in mothers: intergenerational effects and the role of reorganization
Source: Front Psychol. 2014 Sep 1;5:966. doi: 10.3389/fpsyg.2014.00966 (PMC4150444; doi:10.3389/fpsyg.2014.00966)
Supplement: Supplementary file 1 [file DataSheet1.DOCX]

***Supplementary Material***

**Unresolved Trauma in Mothers: Intergenerational Effects and the Role of Reorganization**

**Udita Iyengar**^1,2,3^**, Sohye Kim**^1,2,4^**, Sheila Martinez**^1,2^**, Peter Fonagy**^2,3,4^**, and Lane Strathearn***^1,2,3,4,5^

^1^Children’s Nutrition Research Center, Baylor College of Medicine, Department of Pediatrics, Houston, TX, USA ^2^Attachment and Neurodevelopment Laboratory, Baylor College of Medicine, Houston, TX, USA

^3^Research Department of Clinical, Educational, and Health Psychology, University College London, London, United Kingdom

^4^Menninger Department of Psychiatry and Behavioral Sciences, Baylor College of Medicine, Houston, TX, USA

^5^The Meyer Center for Developmental Pediatrics, Texas Children’s Hospital, Houston, TX, USA

***Correspondence:** Dr. Lane Strathearn, Baylor College of Medicine, Children’s Nutrition Research Center, Attachment and Neurodevelopment Laboratory, 1100 Bates Street, Suite 4004, Houston, TX, 77030, USA.

lanes@bcm.edu

1. **Supplementary Data**

**Criteria for determining a Reorganizing speaker**

Reorganizing speakers are actively changing their understanding of their past and current experience (Crittenden and Landini, 2011). The following list contains hallmark features of reorganization in the direction of balance.

1. A history consistent with a non-secure self-protective strategy for regulating relationships.

2. Use of a non-secure self-protective pattern of discourse together with the speaker’s at least implicit awareness of the strategy and some of the reasons for it.

3. A cooperative relationship with the interviewer in which the speaker actively engages in the review of his or her history *for the purpose of finding meaning.*

4. Self-monitoring such that some discrepancies and slips into the old self-protective pattern are noticed or corrected.

5. Behavioral evidence in the discourse that the dominant pattern is being reversed.

6. A reflective stance with evidence of a) taking in and using new information to arrive new understandings, b) weighing and considering alternative perspectives, or c) active efforts to tie past and present together in a psychologically sound manner.

7. Overt awareness that appearances are not always synonymous with reality.

8. Statements of self-efficacy that are supported with evidence.

9. Overt articulation of change, including past misunderstandings and reasonable current understandings, change in perspective, or description of active effort to change and the process of change.

10. Concrete evidence of change in the way the speaker lives his or her life.

1. **References**

Crittenden, P., & Landini, A. (2011). *Assessing adult attachment: A dynamic-maturational approach to disourse analysis*. New York, NY: W. W. Norton & Company.
